# CO_2_–Binder Reaction Mechanisms in Geopolymer Wellbore Cements: Alternatives to API Class G Cement in CO_2_-Rich Environments (CCS)

**DOI:** 10.3390/molecules31040620

**Published:** 2026-02-10

**Authors:** Omer Mohamed Bakri, Ahmed Abdulhamid Mahmoud

**Affiliations:** 1 Department of Petroleum Engineering, King Fahd University of Petroleum & Minerals, Dhahran 31261, Saudi Arabia; g202409440@kfupm.edu.sa; 2 Center for Integrative Petroleum Research, King Fahd University of Petroleum & Minerals, Dhahran 31261, Saudi Arabia

**Keywords:** geopolymer cement, alkali activation, wellbore sealing, carbon capture and storage (CCS), supercritical CO_2_, CO_2_-induced degradation, permeability, microstructure, zonal isolation, API class G cement

## Abstract

API Classes of cement are susceptible to three major problems: carbonation, decalcification, and increased porosity of cement sheaths in CO_2_-rich environments. These degradation pathways in American petroleum institute (API) Class/ordinary Portland cement (OPC) systems are well documented in laboratory and field observations for CO_2_-rich wellbore service. In contrast, while geopolymer/alkali-activated binders have been increasingly studied as alternatives, the evidence remains distributed across different precursor chemistries, exposure conditions, and test protocols, and a consolidated, mechanism-based synthesis specific to CO_2_ sequestration wells is still limited. Accordingly, this article presents a critical, narrative (non-systematic) review that synthesizes published laboratory and field studies on geopolymer/alkali-activated binders for CO_2_ sequestration wells, with emphasis on permeability, strength retention, and microstructural stability under CO_2_-rich exposure. The main outcome of this review is a mechanism-based synthesis that links CO_2_–binder reaction pathways (gel chemistry/phase evolution) to pore-network and transport changes, and consolidates quantitative performance benchmarks (permeability and strength retention) relative to API Class G/OPC, while defining the key validation gaps for qualification (HPHT, cyclic/tensile integrity, mixed fluids, and long-term monitoring). Laboratory tests have already demonstrated that geopolymer samples have ultralow permeability and preserve 90% of their strength after being treated with supercritical CO_2_ concentrations, while OPC loses its strength and produces macropores causing substantial growth of cement sheath porosity. Microstructural studies have shown that geopolymers do not contain portlandite but only N–A–S–H/C–A–S–H gels with low Ca content in concentrations high enough to create N–A–S–H/C–A–S–H gels, but do not suffer from multi-zone carbonation, as occurs for OPC concrete. Key challenges being tackled include slurry rheology, setting control and variability of precursors by designed admixture use and new performance specifications for higher-quality geopolymers. On the whole, geopolymers emerge as a sustainable and reliable alternative to traditional well cementing techniques for their sustainability well integrity.

## 1. Introduction

CO_2_ geological sequestration delves challenging requirements onto the wellbore materials. A central challenge for carbon capture, utilization and storage (CCUS) is preventing and monitoring long-term CO_2_ leakage through wells, since small losses of zonal isolation can create persistent migration pathways along the cemented annulus [1]. Conventional oil-well cement, normally Class G (OPC), is susceptible under the CO_2_-rich subsurface environment. Experimental studies of [2,3,4] consistently report that OPC is subject to carbonation reactions resulting in concentric alteration zones: an unaltered core, a decalcified region depleted in portlandite, a calcite (CaCO_3_)-enriched zone, and an outer leached silica zone. This degradation increases porosity and permeability, while reducing compressive strength and elastic modulus and directly undermining long-term well integrity. Field surveys have confirmed this vulnerability, as [5] noted that the majority of well integrity failures in CO_2_ injection projects were linked to OPC sheath degradation. Ref. [6] further show that sour gas environments (CO_2_ + H_2_S) accelerate these degradation pathways.

On the other hand, geopolymer cements are alkali-activated aluminosilicate cementing materials based on industrial by-products like fly ash, slag, volcanic ash, red mud, or granite powder. These cements do not contain any portlandite, in contrast to OPC, which makes them naturally resistant to carbonation and acid attacks [7].

Another series of studies by [8] showed that fly ash-based geopolymers in supercritical CO_2_ environments have permeability 100–1000 times lower than OPC, with substantial retention of their compression strength. Ref. [9] showed no loss in strength after exposure to CO_2_-saturated brine at 90 °C, in contrast to marked decreases in OPC samples, with many samples undergoing pronounced degradation due to formation of microcracks. These findings have since been validated in a meta-analysis by [10], indicating substantial retention of ~90% for geopolymer samples in comparison with ~60% retention for OPC samples after exposure to CO_2_. Another advantage provided by geopolymer cementing is its inherent sustainability properties. Geopolymers have now emerged as promising green cements with low carbon footprints, requiring only low energy for clinkerless production, soon to have the potential to lower the carbon footprint associated with cementing operations by 60–85% [11]. Their success in real-world applications has recently been documented in [12,13] to have successfully employed geopolymer-based cementing systems (EcoShield(R)) for CO_2_ emission reductions to the tune of ~85%, in comparison to OPC cementing systems. Their favorable properties have allowed these materials to be chosen exclusively for applications in CO_2_ sequestration injection wells, for their long-term stability.

Although this review focuses on CO_2_-rich service because it is central to CCS/CCUS well integrity and is the dominant exposure condition in many storage scenarios, real subsurface wells may experience mixed-fluid environments. These can include sour components (H_2_S) and brines containing high salinity and divalent ions (e.g., Ca^2+^, Mg^2+^, Fe^2+^), which may introduce additional reaction pathways and transport effects beyond CO_2_-driven carbonation. Compared with the CO_2_-focused literature, systematic durability data for geopolymer/alkali-activated wellbore binders under sour gas and mixed-fluid conditions remain limited. This represents an important knowledge gap, and future qualification efforts should evaluate geopolymer performance under combined CO_2_–brine–H_2_S exposures and relevant cycling to better reflect field conditions.

Despite the encouraging findings reported for geopolymer/alkali-activated binders, the existing literature remains heterogeneous and not yet fully systematized for CO_2_-well applications. Studies differ widely in precursor chemistry (fly ash/slag/natural pozzolans), activator composition (alkali type, dosage, silicate modulus), curing history, and exposure conditions, which complicates direct comparison and generalization. A key scientific deficit is the limited consolidation of CO_2_–binder chemical mechanisms, including dissolution/precipitation reactions, carbonate/bicarbonate interactions, and the resulting evolution of N–A–S–H/C–A–S–H gel chemistry and phase assemblage, and how these molecular-scale processes translate into pore-network evolution, permeability, and long-term sealing integrity. Consequently, a mechanism-focused synthesis and clearer mapping of what is well established versus what remains uncertain (e.g., HPHT behavior, cyclic/tensile integrity, mixed-fluid exposure, and long-term field monitoring) are still needed to support qualification and wider deployment.

This review integrates and critically synthesizes published laboratory studies and available field reports on geopolymer/alkali-activated binders for CO_2_-rich wellbore environments. The goal is to evaluate the evidence for durability and sealing performance relative to API Class G/ordinary Portland cement (OPC) under CO_2_ exposure, connect reported performance to the governing reaction mechanisms and microstructural evolution, and clearly identify the main barriers to deployment and remaining knowledge gaps. In particular, we highlight validation needs relevant to real storage wells, including performance under high-pressure/high-temperature conditions, tensile and cyclic-load integrity, mixed-fluid exposure (e.g., CO_2_–brine–H_2_S), and the limited availability of long-term field monitoring data. We then synthesize the literature into five themes: geopolymer chemistry and composition, applications in CO_2_-rich formations, mechanical and durability performance, microstructural analysis, and challenges and outlook.

## 2. Review Methodology and Scope

This article is a critical, narrative (non-systematic) review. The literature was identified using structured searches in Scopus, Web of Science, ScienceDirect, Google Scholar, and petroleum-engineering repositories including One Petro/SPE proceedings. Search terms were combined using Boolean operators and included the following: geopolymer, alkali-activated, oil-well cement, wellbore cement, zonal isolation, CO_2_ sequestration, carbon capture and storage (CCS), supercritical CO_2_, carbonation, decalcification, permeability, HPHT, thickening time, rheology, casing bond log (CBL/VDL), cement–casing interface, and one-part geopolymer. Priority was given to peer-reviewed journal papers and well documented conference papers reporting experimental measurements (mechanical performance, permeability/transport, and microstructural characterization) under CO_2_-rich or related downhole conditions. Additional sources were included to capture field deployment reports and standards context where available. The synthesis is organized thematically to connect observed performance to governing mechanisms and to identify remaining qualification needs for CO_2_-rich wellbore service.

This review is intended for wellbore integrity and cementing practitioners (petroleum and drilling engineers), CCS/CCUS project teams, and materials/cement researchers evaluating low-carbon alternatives to API Class G/OPC for CO_2_-rich environments. It is also relevant to operators and regulators seeking performance-based evidence and knowledge gaps that inform qualification and monitoring expectations for CO_2_ storage wells.

## 3. Geopolymer Chemistry and Composition

**Figure 1:**
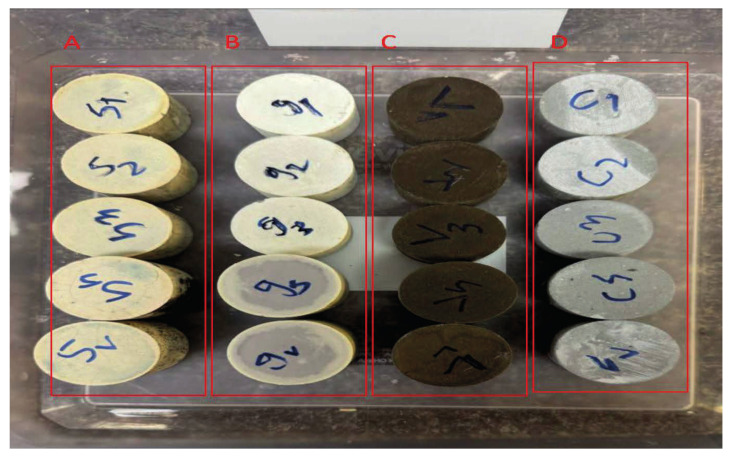
Representative cylindrical specimens of geopolymer/alkali-activated and API Class G/OPC systems prepared for CO_2_-rich exposure and integrity testing, A: slag, B: glass fiber, C: volcanic scoria, and D: OPC.

**Figure 2:**
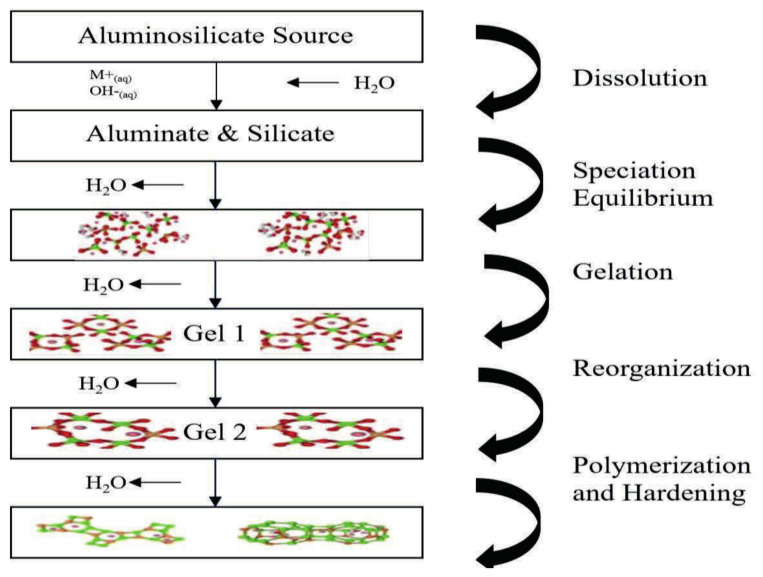
Schematic illustration of the geopolymerization reaction mechanism modified after [17].

Geopolymers are alkali-activated aluminosilicate binders that differ fundamentally from Portland cement in both composition and binding phase [14,15]. In a typical geopolymer, aluminosilicate source materials rich in SiO_2_ and Al_2_O_3_, such as low-calcium fly ash, ground granulated blast-furnace slag, calcined clays (e.g., metakaolin), volcanic ash, silica fume, or bauxite residue (red mud) are mixed with an alkaline activator (often NaOH, KOH, and/or sodium silicate). Figure 1 shows cylindrical specimens of different precursors. Recent studies also highlight mine tailings and construction and demolition waste (CDW) as additional aluminosilicate-rich feedstocks for geopolymer production, supporting circular-economy pathways beyond conventional fly ash/slag supply [16]. The alkaline solution dissolves reactive silica and alumina, initiating polymerization into a hardened, amorphous three-dimensional network of Si–O–Al bonds, details of the geopolymerization reaction is illustrated in Figure 2.

The chemical mechanism involves three stages: dissolution of reactive species, transport and orientation of dissolved ions, and polycondensation A simplified reaction which can be represented by Equation (1).

(1) $${\mathrm{Al}}_{2}O_{3}\cdot 2{\mathrm{SiO}}_{2}\cdot 2H_{2}O+\mathrm{NaOH}\backslash {\mathrm{Na}}_{2}{\mathrm{SiO}}_{3}\backslash \mathrm{KOH}\to M_{n}{\left[-\left({\mathrm{SiO}}_{2}\right)\_z-{\mathrm{AlO}}_{2}\right]}_{n}\cdot wH_{2}O$$

The general chemical formula of a fully reacted geopolymer can be expressed as M_n_ [−(SiO_2_) z-AlO_2_]_n_ · wH_2_O, where M is a monovalent cation (Na^+^, K^+^, or sometimes Ca^2+^ in a charge-balancing role), *z* is the Si/Al ratio, and *w* is the degree of hydration (all of which depend on precursor chemistry).

The dominant binding phase in fly ash-based systems is sodium aluminosilicate hydrate (N–A–S–H gel), while slag-rich blends also form calcium aluminosilicate hydrate (C–A–S–H). The latter contributes to early strength but introduces calcium that can carbonate. Unlike OPC, geopolymers contain negligible portlandite (Ca (OH)_2_) or other readily soluble Ca-phases, which explains their superior durability: with little free calcium to leach or carbonate, the aluminosilicate network remains intact under CO_2_-rich or acidic conditions [18].

Another distinction is that geopolymerization can incorporate CO_2_ into stable phases rather than producing it as a by-product. OPC hydration liberates Ca (OH)_2_ that later carbonates, weakening the matrix, while geopolymer systems can precipitate benign carbonates or aluminosilicates without compromising integrity. Ref. [19] showed that fly ash geopolymers exposed to 1–5 M H_2_SO_4_ at 180 °C did not degrade but instead formed stable natroalunite crystals, retaining strength. Similarly, refs. [8,9] observed only minor carbonate precipitation limited to sample surfaces in CO_2_-saturated brines, with no deep carbonation front, as seen in OPC.

Precursor chemistry strongly influences performance. Low-calcium fly ash yields slower-setting but highly acid- and CO_2_-resistant binders. Slag additions accelerate early strength and densify the microstructure through additional C–A–S–H gel formation, dramatically reducing permeability. Ref. [8] found that adding 15% slag to a fly ash geopolymer reduced CO_2_ permeability by nearly three orders of magnitude compared with Class G OPC. However, high slag contents may reintroduce carbonation or sulfate susceptibility, so blends with moderate slag (<15%) often achieve the best balance of strength and durability.

Red mud has been studied as a supplementary precursor: Ref. [20] concluded it is unsuitable alone but effective in 10–20% blends with fly ash or slag, especially if pretreated to adjust its soda and alumina contents [21]. Innovative natural feedstocks are also being explored, such as granite fines [22] and volcanic ash [23], both showing promise when combined with appropriate activators or polymer modifiers.

Mix design requires the tuning of activator molarity, silicate dosage, solid-to-liquid ratio, and curing schedule. Higher alkali content increases reaction extent and strength up to an optimum; beyond that, excess alkali can cause gel exudation or efflorescence [24]. Optimum Na_2_O/Al_2_O_3_ ratios of ~0.6–0.7 have been reported for fly ash geopolymers. Sodium silicate solutions (“waterglass”) improve early strength but reduce workability and shorten setting times. One-part (“just-add-water”) systems, such as those demonstrated [25,26], replace liquid activators with powdered chemicals like Na_2_SiO_3_, simplifying field handling at a slight cost to early strength.

Density adjustments make geopolymers adaptable to deep or shallow wells. Heavy-weight formulations can be achieved with additives like hematite, barite, or ilmenite: for example, ref. [27] produced an 18.5 ppgs (pounds per gallon) geopolymer. Ref. [28] further stabilized high-density slurries by partially substituting hematite with fine Micromax particles, preventing settling and ensuring uniform properties. Lightweight geopolymers, conversely, can be formulated with cenospheres, nano-silica, or gas foaming. Ref. [29] reviewed lightweight cement design principles (target densities ≈ 1.2–1.5 g/cc) that directly apply, while [30] showed that adding cenospheres and nano-silica reduces density and refines pore structure in cementitious matrices.

Additives and admixtures provide further tuning. Retarders are critical for high-temperature, high-pressure applications: Ref. [22] successfully extended set times in a granite-based geopolymer using Zn–K salts. Rheology modifiers like attapulgite clay improve suspension stability, while polycarboxylate superplasticizers can reduce viscosity if they are compatible with the high-alkali environment. (Not all OPC admixtures translate effectively; for instance, lignosulfonates lose efficacy in geopolymer systems).

Polymer additives have been shown to enhance toughness: Ref. [23] used an epoxy resin to improve bonding and strength, while [31] demonstrated in OPC that polypropylene fibers reduce cracking, suggesting similar benefits in geopolymers. Nanomaterials like graphene oxide [32] have also been shown to refine microstructure and reduce fluid loss.

In summary, geopolymer chemistry provides a tunable binder system in which industrial waste-derived aluminosilicates and alkali activators form low-Ca, highly cross-linked gels with intrinsically low permeability and exceptional resistance to CO_2_ and acid. Through precursor blending, activator optimization, and tailored admixtures, geopolymers can be engineered for both heavy-weight and lightweight well cementing applications, offering a compelling alternative to OPC in CO_2_ sequestration wells.

## 4. Applications in CO_2_-Rich Subsurface Environments

Laboratory simulations and trials: Early studies established that fly ash-based geopolymers can meet API performance targets while withstanding aggressive CO_2_ exposure. Pioneering work by [8,33] showed that, in triaxial cells at 80 °C and 15 MPa CO_2_, optimized fly ash/slag geopolymers exhibited apparent CO_2_ permeabilities that are drawn from experimental measurements using pressure-transient/undrained permeametry, on the order of 10^−21^–10^−20^ m^2^ (≈0.0005–0.02 μD)—about 100–1000× lower than neat Class G OPC, while maintaining ~20–30 MPa UCS after exposure. Companion OPC samples dropped below 10 MPa and developed fracture networks. Independent tests corroborate this resilience: Ref. [34] reported favorable rheology, low filtrate loss, and higher 24 h strength than Class G; ref. [9] observed negligible strength loss and intact microstructure after aging in CO_2_-saturated brine at 90 °C; and ref. [35] found better integrity and fluid loss control than Class G at elevated temperature, though pumpability (thickening time) still required optimization.

Precursor diversity and regionalization: A wide range of precursors have been explored to leverage local industrial by-products. Class F fly ash [9,10] and Class C fly ash [34] have been used, as well as slag or fly ash/slag blends [25,36], metakaolin/bentonite mixes [37], volcanic ash [23], granite fines [38], and red mud [20]. For example, Hajiabadi et al. tailored a granite-based geopolymer with K-based activators, reaching >40 MPa compressive strength and remaining intact in CO_2–_brine at 150 °C [38]. Alanqari et al. formulated a volcanic ash geopolymer with NaOH/silicate plus an epoxy additive that set in ~8 h at 60 °C and was pumpable in practice [39]. This adaptability enables geopolymers to match regional feedstocks and specific well conditions.

Density tailoring, heavy-weight and lightweight: For deep wells, heavy-weight geopolymer slurries have been achieved by blending conventional weighting agents. Ref. [40] produced ≥18 ppg systems by combining hematite (SG ~ 5) with fine Micromax, suppressing particle settling to yield homogeneous slurries that reached ~30 MPa compressive strength at 24 h (meeting typical high-pressure/high-temperature (HPHT) requirements). Conversely, lightweight designs for weak formations or thermal insulation draw on foaming and hollow microspheres. Ref. [41] outlined design principles for lightweight cement (1.2–1.5 g/cc) that apply to geopolymers; ref. [30] showed that adding cenospheres and nano-silica lowers density while refining pore structure, an approach now applied in geopolymer formulations. Ref. [36] even demonstrated a cold-setting (7–15 °C) alkali-activated slag composite with ~5% resin and a coupling agent, improving early strength and casing bond versus OPC in deepwater conditions.

Field-readiness one-part systems: To simplify handling and eliminate bulk caustic liquids, “one-part” (just-add-water) geopolymer formulations have been developed. Ref. [25] combined slag with pretreated paper sludge and powdered NaOH, achieving >40 MPa compressive strength at 28 days. Ref. [26] used natural pozzolans with solid Na-silicate to achieve >20 MPa at 28 days. These dry blends mirror OPC job logistics (dry silo + mix water), improving HSE and deployment speed. However, the published dataset for one-part systems under deep-well HPHT conditions (e.g., ~30 MPa and >200 °C) remains limited compared with conventional cement systems and two-part geopolymer formulations. Therefore, their applicability to extreme CO_2_ storage wells should be treated as formulation-specific and validated with representative HPHT curing/aging (including CO_2_/brine exposure and cycling) before broad generalization [26,42,43,44]. Any reactivity shortfall is countered via highly reactive components (e.g., slag, quicklime) and optimized curing.

- **Field deployments:** Multiple field trials confirm the real-world viability of geopolymer well cement.
- **Offshore CCS (Malaysia):** Ref. [13] successfully placed a fly ash geopolymer section in a CO_2_ injection well. Bond logs and pressure tests showed excellent zonal isolation, validating offshore mixing and pumping. However, the published information mainly reflects initial/early post-installation verification, and the open literature provides limited details on long-term follow-up monitoring timelines (e.g., repeated CBL/VDL, sustained annular pressure trends, or periodic integrity tests). Therefore, this case should be interpreted as strong evidence of field feasibility and short-term integrity, while multi-year monitored datasets are still needed to confirm long-term suitability.
- **Onshore, cement-free system:** Refs. [12,45] reported the first full-scale primary cementing using a proprietary cement-free geopolymer (EcoShield^®^, Gilbert, AZ, USA)—~100 m^3^ mixed and pumped with standard equipment. Logs confirmed competent sheaths, and the operation’s CO_2_ footprint fell by ~85% versus an equivalent OPC job.
- **Wider adoption signals:** Ref. [46] urged industry uptake (projecting ~80% emissions cuts). Ref. [11] summarized multiple jobs (primary and remedial) across regions involving tens of cubic meters of geopolymer, with performance on par with OPC.
- **Analogous high-T wells:** In geothermal simulations, ref. [47] found an alkali-activated sealant outperformed OPC at up to 300 °C, underscoring suitability for supercritical CO_2_ reservoirs.

Table 1 below provides a comparative summary of the behavior and performance of Portland cement and geopolymer cement systems under CO_2_ exposure, highlighting key differences in chemical stability, permeability, mechanical integrity, and environmental impact.

Quantitative synthesis across studies. Across triaxial and CO_2_–brine aging datasets reviewed here, geopolymer/alkali-activated wellbore binders commonly report apparent CO_2_ permeability on the order of 10^−21^–10^−20^ m^2^ (study- and method-dependent) with high compressive strength retention frequently approaching ~90% after CO_2_-rich exposure, whereas API Class G/OPC systems more often exhibit pore coarsening/cracking and materially lower strength retention under comparable exposure severity. These quantitative benchmarks, when interpreted alongside microstructural observations (limited deep carbonation fronts in low-Ca systems and localized carbonate precipitation), provide a consolidated evidence base for why geopolymers can maintain sealing performance in CO_2_-rich environments.

Geopolymers have progressed from controlled lab trials to credible field implementations, covering the full oil-well density spectrum while maintaining very low permeability and strong strength retention after CO_2_ exposure. Their compatibility with standard cementing hardware, availability as one-part dry blends, and successful onshore/offshore demonstrations position geopolymers as a practical, lower-carbon alternative to OPC for CO_2_-rich subsurface environments.

## 5. Mechanical and Durability Performance

For any cementing material, compressive strength, tensile capacity, and durability under aggressive downhole conditions are critical. OPC is well known to lose strength and stiffness after carbonation: Refs. [53,54] observed progressive decreases in elastic modulus and UCS for OPC under supercritical CO_2_ (scCO_2_). In contrast, geopolymer systems consistently demonstrate higher strength retention under similar conditions.

Ref. [34] reported that their Class C fly ash geopolymer achieved UCS values exceeding those of Class G OPC after 24 h, while also maintaining low fluid loss. Ref. [9] confirmed negligible UCS loss in fly ash geopolymers after exposure to CO_2_-saturated brine at 90 °C, in stark contrast to OPC samples that cracked and weakened. Ref. [10], in a meta-analysis, consolidated these findings: fly ash geopolymers typically retain ~90% of their compressive strength after CO_2_ exposure, compared with ~60% for OPC. Ref. [33] also demonstrated that, under triaxial stress conditions, geopolymer samples exhibited stable compressive strength and low deformation, resisting the radial cracking that [55] predicted for OPC under cyclic injection loads.

Absolute strength values vary with precursor chemistry and curing. Ref. [38] achieved compressive strengths above 70 MPa in a granite-based geopolymer optimized with a Zn–K retarder at 150 °C, while [56] noted strengths of 20–30 MPa accompanied by slight expansion beneficial for zonal isolation. Ref. [36] showed that adding a resin and coupling agent to an alkali-activated slag composite improved early-age strength in deepwater conditions (7–15 °C). These examples illustrate that, with proper formulation, geopolymer systems can meet or exceed the mechanical benchmarks of OPC.

Tensile strength and ductility are often the Achilles’ heel of brittle binders where modification strategies are being explored. Ref. [33] measured geopolymer tensile strength at ~10–15% of UCS (like OPC) and emphasized the need for improvement. Ref. [31] addressed this in OPC by incorporating 0.125% polypropylene fibers. Their fiber-reinforced OPC showed carbonation rates reduced by 31%, permeability lowered by ~30%, and compressive and tensile strengths increased by 34–51% and 19–27%, respectively, after 10–20 days in CO_2–_brine at 130 °C. While demonstrated on OPC, similar fiber additions to geopolymers are expected to improve toughness and crack resistance. Ref. [57] demonstrated in concrete that hybrid steel fibers mitigated strength losses caused by using recycled brick powder; analogous approaches could be applied to brittle geopolymer matrices.

Polymer and nanomaterial modifications have also proven effective; [23] showed that an epoxy resin not only extended working time in a volcanic ash geopolymer but also enhanced bonding and UCS. Ref. [32] incorporated 0.2% graphene oxide nanosheets into a fly ash geopolymer, achieving ~25 MPa UCS at 120 °C and reducing fluid loss by ~23%. Ref. [58] demonstrated in OPC that a triazine-based polymer increased strain-at-failure and reduced young’s modulus, highlighting polymer modification as a transferable strategy. Together, these results indicate that polymers, fibers, and nanomaterials can make geopolymer systems more ductile and resilient, reducing the risk of brittle failure under pressure and thermal cycles.

Durability under aggressive fluids is perhaps the clearest advantage of geopolymers. Ref. [19] exposed fly ash geopolymer pastes to hot sulfuric acid (up to 220 °C) and observed not degradation but slight strength gains due to the precipitation of natroalunite crystals. Similar acid and sulfate resistance has been reported by [59] and others, showing that geopolymers lack the calcium aluminates and portlandite phases that OPC converts into expansive or leachable products. Ref. [60] confirmed that adding supplementary cementitious materials (SCMs) to OPC reduces portlandite content and carbonation susceptibility; geopolymers, composed almost entirely of SCM-like precursors, inherently possess this durability advantage.

In terms of permeability and porosity, ref. [8] measured CO_2_ permeabilities in fly ash geopolymers on the order of 10^−5^–10^−3^ mD, compared with ~1–10 mD for OPC. Refs. [9,10] confirmed that, even after prolonged CO_2_ exposure, geopolymers retain ultralow permeability, whereas OPC permeability increases by orders of magnitude once carbonated layers collapse. This intrinsically low transport property, often further improved by slag addition [61], makes geopolymers ideal barrier materials.

High-temperature performance is another strong point. Ref. [47] tested alkali-activated aluminosilicates under geothermal conditions up to 200 °C, finding superior stability and strength retention compared with OPC. Ref. [19] similarly noted that geopolymers remained stable at 180–220 °C in acid. These results indicate that geopolymers not only resist CO_2_ chemically but also withstand HPHT conditions in which OPC suffers strength retrogression. To strengthen the claim for up to 220 °C performance and HPHT CO_2_ contexts, more direct studies like [62] are needed with explicit mechanical tests under CO_2_+ pressure at such temperatures.

In sum, geopolymer systems exhibit compressive strengths on par with OPC, far greater retention of strength under CO_2_ attack, ultralow and stable permeability, and exceptional acid and thermal resistance. While ductility and tensile capacity remain areas for improvement, the use of polymers, fibers, and nanomaterials has already shown promising enhancements. These attributes support the suitability of geopolymers as durable wellbore sealants for long-term CO_2_ sequestration.

## 6. Microstructural Analysis

In CO_2_-rich wellbore environments, degradation or stabilization is governed by coupled transport and chemical reactions. Dissolved CO_2_ forms carbonic species (H_2_CO_3_/HCO_3_^−^/CO_3_^2−^) that reduce pore solution pH and can drive dissolution–precipitation reactions. In API Class/OPC systems, portlandite-rich hydration products are highly susceptible to decalcification, producing zoned alteration and porosity increase [63,64,65]. In contrast, geopolymer/alkali-activated binders are dominated by aluminosilicate gels (N–A–S–H) and/or calcium-aluminosilicate gels (C–A–S–H), with little to no portlandite, so the dominant CO_2_ pathways shift from portlandite depletion to (i) gradual changes in gel chemistry, (ii) precipitation of carbonate phases where Ca is available, and (iii) modification of pore connectivity rather than rapid formation of distinct alteration fronts [62,66,67].

From a microstructural standpoint, three competing outcomes are frequently reported depending on chemistry and exposure conditions. First, carbonate precipitation (particularly in Ca-bearing systems or where Ca is mobilized) can locally fill pores and reduce permeability (“pore-blocking”), which is consistent with observations of densification and reduced transport in some studies. Second, prolonged exposure and alkali redistribution may alter gel crosslinking and bound-water content, potentially contributing to shrinkage-related microcracking under restraint and thermal/pressure cycling. Third, where dissolution dominates over precipitation (e.g., aggressive brines, sustained low pH, or unfavorable gel composition), microstructure can exhibit pore coarsening and connectivity increase, which directly elevates permeability and leakage risk [68,69,70].

Accordingly, the microstructural evidence discussed in this section (SEM/EDS, XRD/FTIR, and porosity/permeability measurements) is interpreted in relation to these mechanisms: changes in gel chemistry and phase assemblage indicate reaction progress, while pore size distribution and crack development determine transport. This mechanistic framing enables more consistent comparison across different precursor/activator systems and clarifies why compressive strength alone is insufficient, because leakage pathways are governed by pore connectivity and crack/interface integrity.

Microstructural evidence helps explain why geopolymers retain strength and tightness under CO_2_ exposure while OPC degrades. In OPC, CO_2_-driven alteration advances as a zoned front: an inner decalcified region depleted of portlandite (CH), a carbonate-rich zone where C–S–H is partly converted to calcite/aragonite (temporarily clogging pores), and an outer leached silica layer with coarser, connected porosity. Imaging and chemical mapping at cement–steel interfaces as [7,71,72] show outward calcium depletion and porosity coarsening, alongside steel cement debonding under cyclic loads that promotes micro annuli and leakage paths.

**Figure 3:**
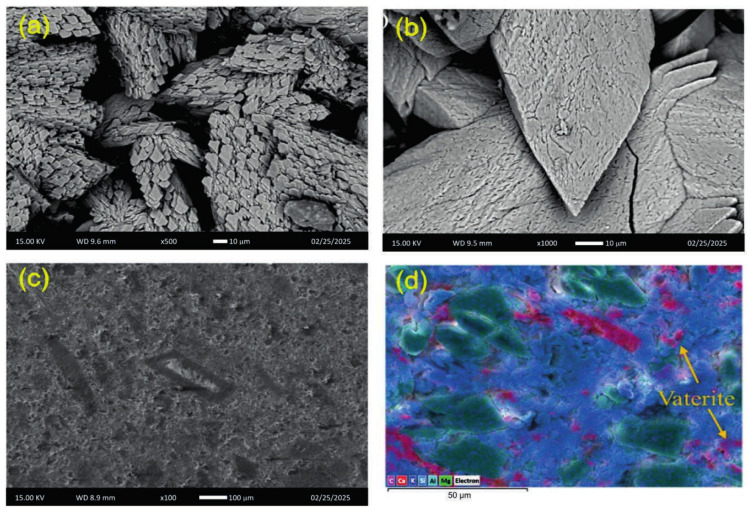
SEM micrographs of the top surfaces [43], (**a**,**b**) at the inlet of the specimens. (**c**,**d**) from the middle portion of the specimens.

Geopolymers lack a CH reservoir and are dominated by N–A–S–H (and, when slag is present, C–A–S–H) networks that are far less reactive with carbonic acid. After aging fly ash geopolymers in CO_2_-saturated brine at ~90 °C, studies like [43] report that distinct rhombohedral calcite crystals are prominently observed in the geopolymer system after exposing to CO_2_, occupying and partially occluding the pore spaces within the geopolymer matrix. This secondary mineralization contributes to a reduction in porosity, and a potential enhancement of the sealing capacity of the material, consistent with the negligible loss measured. Across micro-CT and mercury intrusion porosimeter datasets, geopolymer pore structures remain mesopore-dominated and stable, whereas OPC evolves toward coarser, connected porosity as carbonated “skin” layers crack or spall. Figure 3 shows SEM micrographs of geopolymers cement systems.

When small amounts of calcium-bearing phases are present in geopolymers (e.g., from minor slag content), the carbonation products are typically limited and can be benign or even self-sealing. Small quantities of calcite or dawsonite may precipitate at exposed surfaces or within gel pores, locally refining the porosity without undermining the matrix. An extreme analog under hot acid conditions was observed by [19]: fly ash geopolymers exposed to 180–220 °C H_2_SO_4_ developed infillings of natroalunite crystals, with XRD confirming new stable sulfate–aluminosilicate phases and no deleterious gel dissolution in sharp contrast to OPC’s conversion of C–S–H into expansive gypsum/ettringite and its severe gel decalcification that cracks and weakens the matrix.

Mix design and admixtures can strongly influence microstructure. Using an optimal ~0.6 wt% Zn–K retarder, a granite-based geopolymer [38] formed a dense, interconnected gel matrix and exhibited progressive recrystallization to stable silicate phases. By contrast, under- or over-retardation left unreacted grains embedded in an amorphous gel with a porous, inhomogeneous texture, correlating with reduced strength and ductility. Epoxy-modified volcanic ash geopolymers [39] and GO-modified fly ash systems [73] similarly show denser matrices and improved interfacial cohesion, matching the lowered fluid loss and higher UCS observed at the macroscale.

**Figure 4:**
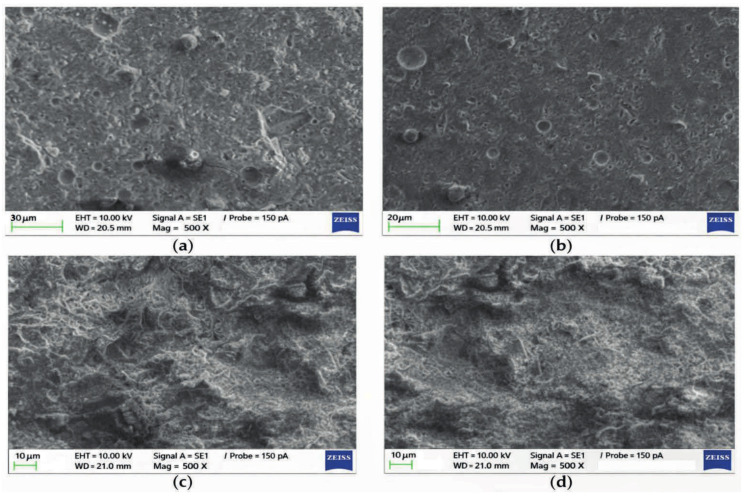
SEM photos (**a**) geopolymer’s surface before carbonation, (**b**) geopolymer’s surface after carbonation, (**c**) OPC before CO_2_ exposure, (**d**) OPC after CO_2_ exposure [74].

Figure 4 shows SEM micrographs from Yigang Lv’s study comparing OPC and fly ash–metakaolin geopolymer paste (F2) before and after 28 days of CO_2_ exposure. Images (a) and (b) show the geopolymer’s surface before and after carbonation, respectively, while (c) and (d) show OPC before and after exposure. The OPC sample develops abundant CaCO_3_ crystals after carbonation, visibly filling pores and densifying the microstructure. In contrast, the low-calcium geopolymer shows minimal visible change aside from minor Na-salt precipitates on the surface, indicating its aluminosilicate gel structure remains largely intact.

Pore structure comparisons via MIP and micro-CT typically show total porosity of ~8–12% in geopolymers (dominated by mesopores <50 nm) versus ~20% in OPC (with more 0.1–1 µm capillary pores). Under CO_2_ exposure, OPC often shows an initial decrease in porosity (due to pore-blocking CaCO_3_ formation) followed by a drastic increase as the altered layer collapses. Geopolymers, by contrast, tend to remain near their initial porosity, with some cases showing pore refinement consistent with their stable ultralow permeability.

Interfaces also matter: OPC commonly develops a porous interfacial transition zone (ITZ) at steel or rock surfaces due to bleed water and CH accumulation, which can preferentially carbonate and debond [72]. Geopolymers frequently exhibit tighter ITZs, less porous and more chemically adherent, consistent with higher measured casing bond strength at low temperature for an alkali-activated slag composite [36] and with excellent bond logs in field deployments [12,13]. Although comprehensive CO_2_-aged ITZ analyses for geopolymers are still limited, available images suggest reduced susceptibility to interfacial carbonation pathways compared with OPC.

Phase identification by XRD reinforces these observations. CO_2_-exposed OPC shows dominant calcite/aragonite peaks and a greatly diminished amorphous hump (indicating C–S–H consumption), whereas geopolymers largely preserve their broad amorphous hump (representing N–A–S–H/C–A–S–H), with only minor surface carbonates like calcite or dawsonite. Notably, dawsonite formation in Na-rich geopolymer systems can self-seal pores by in situ crystallization. Overall, geopolymers undergo minimal crystallographic transformation under CO_2_ compared to OPC’s fundamental phase changes (CH and C–S–H → CaCO_3_ + silica gel).

In summary, observations from SEM, XRD, FTIR, and porosimetry converge on a single mechanism: geopolymers’ low-Ca, highly polymerized gels resist carbonic dissolution and do not develop OPC’s multi-zone damage architecture. Any surface carbonate or sulfate precipitates tend to fill pores rather than dismantle the binder. This microstructural stability directly underpins the geopolymers’ strength retention, ultralow permeability, sustained bonding, and long-term suitability for CO_2-_well sealing.

## 7. Challenges and Limitations

While geopolymers are strong candidates for CO_2_-well sealing, they are not a drop-in replacement for OPC. Several technical and practical gaps must be managed for broad adoption.

Rheology, set control, and pumpability: Geopolymer slurries can be more viscous and thixotropic than OPC, and they are sensitive to activator chemistry. Ref. [35] found that fly ash geopolymers with excellent fluid loss performance still needed thickening time and rheology optimization to meet API pumpability criteria. Chemistry windows can be narrow: excessive NaOH or silicate can trigger rapid gelation or flash set (as noted in high-alkali mixes like [73]). Admixtures must be purpose-built: Ref. [36] showed 5% resin in an alkali-activated slag composite improved early strength and bond at 7–15 °C, whereas >10% created porous, weak matrices. Beyond formulation, rheology models and test methods tuned for OPC (e.g., API thickening time tests, gel strength development profiles) need recalibration for geopolymers. On the practical side, field trials (e.g., [45]) have already shown that mixing and pumping with conventional cementing equipment is feasible—provided chemistry is adjusted for acceptable pump times.

Tensile/fatigue performance and cyclic loading (and fiber efficacy under CO_2_): Compressive strength alone is not sufficient for wellbore integrity, which is often governed by tensile capacity, fracture toughness, and resistance to cyclic thermo-mechanical loading (pressure/temperature cycling) that promotes microcracking, debonding, and microannulus formation [75,76,77]. Although fiber reinforcement is well established for improving crack control in OPC-based systems, direct evidence for fiber-reinforced geopolymer/alkali-activated binders under CO_2_-rich exposure is limited, particularly regarding post-exposure crack-bridging effectiveness and fiber–matrix interfacial durability [78,79,80]. This represents a critical knowledge gap for practical deployment and warrants targeted testing that combines CO_2_/brine aging with cyclic loading, reporting tensile strength, fracture toughness/energy, fatigue crack growth, and permeability evolution in cracked specimens and at cement–casing/cement–formation interfaces.

Raw-material variability and QA/QC: Unlike standardized OPC clinker, geopolymer precursors (fly ash, slag, natural pozzolans) vary in glass content, fineness, alkali levels, and CaO/MgO content, which alters setting and durability. A practical challenge for scaling geopolymer/alkali-activated binders is the intrinsic variability of natural and industrial precursors. For example, fly ash sourced from different coals and combustion conditions can exhibit significant differences in SiO_2_/Al_2_O_3_ ratio, CaO and Fe_2_O_3_ contents, amorphous fraction, fineness, and loss on ignition (LOI)/unburnt carbon, all of which can alter dissolution kinetics, setting behavior, gel chemistry, and ultimately mechanical and transport performance. Similar variability can occur in slag (basicity, MgO, glass content) and volcanic/natural pozzolans (mineralogy and reactive glass fraction). To reduce sensitivity to feedstock variability, future deployment should adopt (i) minimum material specifications and incoming QC (major oxide limits, LOI, fineness, and a proxy for reactive/amorphous content), (ii) blending/co-precursor strategies (e.g., fly ash with slag or metakaolin) to stabilize reactive chemistry and early-age kinetics, and (iii) adaptive formulation by tuning activator modulus and dosage (and liquid/solid ratio) to target consistent workability, setting, and strength development [81,82,83]. In practice, these measures should be coupled with performance-based acceptance tests (e.g., thickening time/workability under job conditions, early strength, and permeability/porosity indicators after CO_2_-rich curing) to ensure reproducible wellbore behavior despite source-to-source precursor variability. Ref. [84] stressed rigorous QA/QC (e.g., XRF, LOI, particle size analysis) and careful blend design (for example, moderating Ca via fly ash/slag ratios) to achieve consistent performance. Red mud is abundant but poorly reactive alone; refs. [20,85] recommend pretreatment and 10–20% blending with fly ash/slag to stabilize its chemistry.

Compatibility with mixed fluids and complex chemistries: Most performance data to date address exposure to CO_2_ and brine; less is known about behavior in H_2_S-bearing environments. Ref. [86] showed that sour gas accelerates OPC degradation; analogous, systematic testing for geopolymers is limited. Interactions with high-salinity brines, divalent cations, and other trace species (Fe, S) merit targeted study. Geopolymer pore solution is highly alkaline at early age; long-term alkali leaching and any impacts on porosity should be monitored, though current evidence suggests leached alkalis re-precipitate as pore-blocking carbonates under downhole conditions.

Supply chain and logistics: High-quality Class F fly ash is regionally constrained and may decline as coal-fired power plants retire; slag availability is also regional. Viable alternatives—volcanic ash [39], granite fines [38], natural pozzolans and one-part blends [26]—require regional adaptation and qualification. Building a reliable supply chain for solid activators (Na_2_SiO_3_/NaOH) and scaling up dry-blend production will be part of commercialization.

Long-term volume stability (creep/shrinkage): Geopolymers typically exhibit low shrinkage and good dimensional stability; however, most published CO_2_ exposure studies on geopolymer/alkali-activated binders report microstructural and transport property evolution over months to a few years, with only limited datasets extending beyond this window. Consequently, long-term (multi-decade) microstructural integrity under sustained CO_2_ exposure cannot yet be confirmed directly from the literature and should not be inferred without caution [87,88,89]. Over longer time scales, additional mechanisms may become relevant, including continued decalcification and secondary phase evolution in Ca-bearing systems, gradual carbonate precipitation/dissolution cycles that can alternately densify or reopen pore networks, and slow redistribution of bound/physically retained water affecting gel structure and shrinkage [90,91]. These processes may be further amplified by wellbore-relevant factors such as temperature and stress cycling, exposure to brine/impurities (e.g., SO_x_/NO_x_), and interfacial pathways at cement–casing and cement–formation contacts. Future work should therefore prioritize long-duration aging (or validated accelerated protocols), coupled chemo-mechanical testing in annular geometries, and reactive transport–mechanics modeling calibrated to measurable microstructural descriptors (pore size distribution, connectivity/tortuosity, gel chemistry, and cracking) to support defensible 20–30-year performance projections [92,93]. OPC is known to form micro annuli from early shrinkage [94]; geopolymers may avoid this, yet creep and autogenous shrinkage should be quantified under confinement. If needed, mild expansion additives (e.g., MgO or CaO—cf., in OPC) or intentionally slight expansive geopolymer formulations [56] can be used to target near-zero net volumetric change.

Standards, qualification, and regulation: API/ISO well cement standards (rooted in OPC; see [95]) do not yet explicitly cover geopolymers. The industry needs performance-based specifications (e.g., minimum strength retention after CO_2_ exposure, maximum post-exposure permeability, bonded interface criteria, HPHT stability protocols) and adapted lab procedures (thickening time tests, rheology profiles at temperature) for qualifying alternative cements. Until such frameworks are published, approvals proceed case-by-case, slowing adoption.

Cost and economics: Although geopolymers leverage low-cost industrial by-products, chemical activators and specialized admixtures add cost. In some regions, geopolymer slurry costs may currently exceed OPC. However, durability-driven OPEX savings (fewer remedial squeeze jobs and leakage repairs) and factors like carbon pricing or credits can shift the economics favorably. Scaling up one-part dry blends in existing cement plants and standardizing precursor supply can also drive costs down [96,97,98].

Training and HSE: Transitioning from OPC will require crew training on different slurry rheology and set behavior, as well as safe handling of high-alkali components (especially in two-part mix systems). One-part dry blends mitigate many HSE concerns, but laboratories and field crews may still need updated protocols (for example, using liners for molds in high-pH mixes) [99].

None of these issues are decisive on their own. The combination of fit-for-purpose admixtures (retarders, polymers, etc.), robust QA/QC, regional precursor strategies, and the emergence of one-part dry-blend logistics together with evolving performance-based standards is already addressing these early hurdles. Continued R&D and field experience will help convert geopolymers from a proven niche solution into a mainstream CO_2_-well cementing option. Table 2 gives a summary of the key challenges and implications for geopolymer wellbore sealing in CO2-rich environments. 

## 8. Outlook and Recommendation

- Performance-based qualification and standardized testing. Progress toward API/ISO-style performance qualification tailored to geopolymers is essential for CO_2_-well service. Beyond compressive strength, qualification should include permeability and strength retention after CO_2_/brine exposure, interface integrity (cement–casing/cement–formation), and representative HPHT stability, with test procedures adapted to geopolymer rheology and setting behavior. Joint industry projects (JIPs) can help harmonize protocols and reporting to enable meaningful cross-study comparisons.
- Integrity under cyclic loading: tensile/fracture focus with practical toughening routes. Wellbore sealing is often controlled by tensile capacity, fracture resistance, and resistance to cyclic pressure/temperature loading rather than compressive strength alone. Accordingly, practical toughening approaches, such as fiber reinforcement and polymer/latex modification should be evaluated specifically for geopolymer matrices under CO_2_-rich aging, with emphasis on post-exposure crack control and permeability evolution.
- Crack management and “self-sealing” concepts (as a wellbore-relevant durability strategy). Because microcracks and micro annuli govern leakage risk, self-healing/self-sealing geopolymer concepts (e.g., microencapsulated healing agents or controlled expansive components) are relevant where they can demonstrably restore sealing capacity after cracking. This should be assessed using wellbore-representative cracked specimens and annular/interface geometries under CO_2_/brine exposure and cycling.
- Bridging operational needs with hybrid systems (only where justified by the well application). In cases requiring rapid-set, specific-density targets, or operational compatibility, hybrid alkali-activated systems (e.g., high fly ash with a limited OPC fraction) may offer practical pathways—provided chemical compatibility is controlled (activator dosage/sequence) and CO_2_ durability is not compromised. These hybrids should be treated as application-specific solutions rather than general replacements.
- Realistic service envelopes: HPHT and mixed-fluid exposure. To avoid overgeneralization, testing should expand to realistic extremes relevant to storage wells, including HPHT conditions (e.g., ~30 MPa and >200 °C where applicable), cyclic loading, and mixed-fluid exposure (CO_2_–brine with divalent ions and/or sour components such as H_2_S). The goal is to define formulation-specific “validated service envelopes” rather than assume universal performance.
- Scalability and field validation: QA/QC and monitored pilots. Deployment requires robust precursor QA/QC and blending strategies to manage variability, including the development of one-part dry blends where appropriate and regionally available feedstocks. Field feasibility should be extended into credibility by pairing pilot jobs with multi-year monitoring plans (e.g., CBL/VDL/ultrasonic logs and pressure integrity tests) and transparent reporting to build 5–10-year datasets.

Table 3 shows how is this review differs from the recent reviews.

## 9. Conclusions

This critical review systematized the current laboratory and field evidence on geopolymer/alkali-activated binders as alternatives to API Class G/ordinary Portland cement (OPC) for CO_2_-rich wellbore environments, with emphasis on the chemical mechanisms controlling durability. Compared with OPC, which degrades through CO_2_-driven carbonation/decalcification and zoned alteration, geopolymers rely primarily on low-Ca aluminosilicate binding gels (N–A–S–H and/or C–A–S–H) and contain negligible portlandite, which fundamentally changes the dominant CO_2_–binder reaction pathways and helps preserve microstructure and transport properties.

Across the reviewed studies, geopolymer systems repeatedly demonstrated ultralow apparent CO_2_ permeability (often reported on the order of 10^−21^–10^−20^ m^2^, i.e., ~0.0005–0.02 μD in representative triaxial datasets) and high compressive strength retention after CO_2_-rich exposure (frequently up to ~90% retention) compared with substantially larger losses and pore coarsening reported for OPC (often ~60% retention in consolidated comparisons). Microstructural observations (SEM/XRD/porosimetry) consistently indicate limited deep carbonation fronts in low-Ca geopolymer matrices; where carbonate phases form, they are often localized and can contribute to pore filling rather than progressive decalcification and weakening, typical of OPC. Field deployments (offshore and onshore demonstrations) further support operational feasibility (mixing/pumping with conventional equipment and evidence of good zonal isolation at installation), while the open literature generally provides limited multi-year monitoring timelines.

Despite these advantages, the review identifies critical barriers to broad qualification and deployment. Key gaps include the following: (i) limited publicly available data for multi-decade integrity (most studies report ≤5 years or shorter) and for post-installation monitoring in field trials; (ii) the need to prioritize tensile/fracture and cyclic-load performance (and to validate whether fiber reinforcement remains effective in geopolymer matrices after CO_2_ exposure); (iii) incomplete evidence for one-part (“just-add-water”) geopolymer performance under deep-well HPHT conditions (e.g., ~30 MPa and >200 °C) relevant to some storage wells; (iv) limited systematic datasets under mixed-fluid conditions, including CO_2_–brine with divalent ions and sour components (H_2_S); and (v) precursor variability and supply chain constraints that require robust QA/QC and performance-based acceptance testing.

Overall, geopolymers emerge as a technically promising and potentially lower-carbon wellbore sealing alternative in CO_2_-rich environments, but “long-term suitability” should be framed as conditional on formulation and validated service envelope. Future progress should couple mechanism-based understanding of CO_2_–binder chemistry with standardized, wellbore-relevant testing (HPHT exposure, cyclic loading, interface integrity) and transparent, longer-term field monitoring to enable performance-based qualification for CCS applications.

## Figures and Tables

**Table 1:** Summary of the main differences between Portland cement and geopolymers cement.

Property	Portland Cement	Geopolymer Cement (Fly Ash/Slag Based)	References
**Composition and Matrix**	Hydration products: Ca-rich phases (portlandite, C–S–H gel); susceptible to acid attack	Alkali-activated aluminosilicate gel; low-Ca content; acid-resistant	[38]
**CO_2_ Interaction**	Carbonates upon CO_2_ exposure; Ca(OH)_2_ + CO_2_→ CaCO_3_; decalcifies C–S–H to silica—weakens structure	Minimal carbonation due to low Ca; stable matrix in CO_2_-rich fluids **Note:** Class C fly ash is typically Ca-rich compared to Class F; this can promote C–A–S–H/mixed N–(C)–A–S–H gels and influence CO_2_ response (e.g., carbonate precipitation and Ca-related alteration pathways)	[33,48]
**Permeability (after CO_2_)**	Permeability increases after CO_2_ exposure; may worsen due to leaching and cracking	Reported ultralow permeability (commonly ~10^−21^–10^−20^ m^2^ in CO_2_-well datasets); often remains low after CO_2_-rich exposure (method/conditions-dependent)	[10,41]
**Mechanical Integrity**	High compressive strength but CO_2_ exposure reduces it; low tensile strength; prone to cracking	Comparable UCS with higher CO_2_ exposure retention; tensile/cyclic integrity remains a key gap—fiber/polymer toughening is promising but requires more CO_2_-aged validation.	[10,38]
**Dimensional Stability**	Undergoes chemical shrinkage during curing (1–2%); forms micro annuli	Minimal shrinkage; better bonding with casing and formation **Note:** Shrinkage is composition- and curing-dependent; slag-rich alkali-activated binders can exhibit higher autogenous shrinkage than OPC in some cases, and may require mix/activator optimization and/or shrinkage-mitigation measures.	[46,49]
**Thermal/Chemical Stability**	Degrades at high temperature; vulnerable to sulfate and acid attack	Stable > 300 °C; excellent resistance to acid and sulfate	[50,51,52]
**Environmental Footprint**	High CO_2_ footprint: ~0.8–1.0 ton CO_2_ per ton of cement	Low CO_2_ footprint; uses industrial waste; up to ~70–85% lower emissions	[13]

**Table 2:** Summary of key challenges and implications for geopolymer wellbore sealing in CO_2_-rich environments.

Challenge/Limitation	Why It Matters for CO_2_-Well Integrity	Practical Mitigation/Validation Need
**Rheology, set control, pumpability**	Controls placement quality, mud removal, and risk of premature gelation/flash set	Formulation tuning (activator modulus/dosage, L/S), fit-for-purpose additives; API-style testing adapted for geopolymers (thickening time, gel strength)
**Raw-material variability (fly ash/slag/natural pozzolans)**	Source-to-source chemistry affects reactivity, gel chemistry, porosity, and durability	Incoming QC (XRF/LOI/fineness/reactive fraction proxies); blending/co-precursor strategies; performance-based acceptance tests
**Mixed fluids and complex chemistries (brine ions, H_2_S)**	Potential synergistic degradation beyond CO_2_-only aging; impacts transport and interfaces	Systematic CO_2_–brine–H_2_S testing; evaluate ion effects (Ca^2+^/Mg^2+^/Fe^2+^) and cycling; monitor alkalinity/ion leaching
**Supply chain and logistics**	Regional precursor availability; activator transport/handling; affects scalability	Regional feedstock strategy; one-part dry blends; standardization of supply and QA/QC
**Long-term integrity (multi-decade)**	Most datasets ≤ 5 years; uncertain pore/phase evolution over 20–30 years	Long-duration aging or validated accelerated protocols; coupled chemo-mechanical exposure; reactive-transport/chemo-mechanical modeling
**Tensile/fracture and cyclic loading; fiber efficacy under CO_2_**	Leakage risk driven by cracking, debonding, micro annuli; compressive strength insufficient	Test tensile strength/fracture energy and fatigue under CO_2_/brine aging; evaluate fiber–matrix interfacial durability and permeability evolution
**One-part geopolymer performance under HPHT**	Limited evidence at deep-well conditions (e.g., ~30 MPa, >200 °C)	HPHT placement + aging validation; phase stability, permeability and strength retention; interface integrity under cycling
**Standards, qualification, regulation**	Current API/ISO frameworks OPC-centered; slows adoption	Performance-based specs for CO_2_ service; adapted lab procedures; define acceptance thresholds for permeability/strength retention and interfaces
**Cost/economics (regional variability)**	Precursor and activator costs/logistics vary; affects adoption	Treat cost parity as qualitative; project-specific TEA considering regional pricing and supply chain
**Training and HSE**	Two-part systems involve high-alkali handling; field execution differences	Preference for one-part where feasible; updated handling protocols and training

**Table 3:** How this review differs from recent reviews.

Review Type	Typical Focus in the Literature	What This Review Adds
Wellbore geopolymer reviews	Parameter optimization; general CO_2_ resistance; well sealant framing	Mechanism → microstructure → permeability framework + qualification envelope
Broad oil-well geopolymer reviews	Feasibility, operational properties, broad durability claims	Quantitative benchmarking + explicit gaps (HPHT, cyclic/tensile, mixed fluids, long-term monitoring)
CCS well cementing technology reviews	Wider CCS/UHS context; degradation mechanisms across binders	Geopolymer-specific CO_2_ reaction pathways tied to transport/sealing integrity
Sustainable alternative binder literature	Alternative binder families	Bridges sustainability with CO_2_-well durability + field qualification needs

## Data Availability

No new data were created or analyzed in this study. Data sharing is not applicable to this article.
